# Statement of Retraction: SOCS5 contributes to temozolomide resistance in glioblastoma by regulating Bcl-2-mediated autophagy

**DOI:** 10.1080/21655979.2026.2667617

**Published:** 2026-05-21

We, the Editors and Publisher of the journal *Bioengineered*, have retracted the following article from publication.

Yu, J., Han, L., Yang, F., Zhao, M., Zhou, H., & Hu, L. (2022). SOCS5 contributes to temozolomide resistance in glioblastoma by regulating Bcl-2-mediated autophagy. Bioengineered, 13(6), 14125–14137. https://doi.org/10.1080/21655979.2022.2081463

Since publication, significant concerns have been raised by a third party about the integrity of the data and reported results in the article. Specifically, concerns were raised about alleged image duplication with similar images from unrelated articles: 

• Figure 2a, panels appear to have been duplicated with similar images in Figure 1e from Wang et al, 2018 (https://doi.org/10.1186/s12943-018-0837-6) and in Figure 1b from Peng et al, 2021 (https://doi.org/10.1155/2021/3672112)

• Figure 4a, panels appear to have been duplicated with similar images in Figure 4B from Sun et al, 2018 (https://doi.org/10.12659/msm.908927) and in Figure 4C from Zhao et al, 2020 (https://doi.org/10.1042/BSR20193431)

• Figure 4c, panels appear to have been duplicated with similar images in Figure 5b from Gao et al, 2019 (https://doi.org/10.1038/s41419-019-1849-x) 

When approached for an explanation, the authors have not responded to our queries and so these serious concerns remain unaddressed.  

As verifying the validity of published work is core to the integrity of the scholarly record, we are therefore retracting the article. The authors have been informed of this decision.

We have been informed in our decision-making by our policy on publishing ethics and integrity and COPE guidelines.  

The retracted article will remain online to maintain the scholarly record and will be digitally watermarked on each page as ‘Retracted’.  

